# Poster Session I - A105 DETERMINING A BENCHMARK FOR THE ST.PAUL’S ENDOSCOPIC COMFORT SCALE (SPECS)

**DOI:** 10.1093/jcag/gwaf042.105

**Published:** 2026-02-13

**Authors:** A Zarrin, C Galorport, H Qian, N Shahidi, R A Enns, J J Telford

**Affiliations:** The University of British Columbia Faculty of Medicine, Vancouver, BC, Canada; St. Paul’s Hospital, Digestive Health Centre, Vancouver, BC, Canada; The University of British Columbia Faculty of Medicine, Vancouver, BC, Canada; St. Paul’s Hospital, Digestive Health Centre, Vancouver, BC, Canada; St. Paul’s Hospital, Digestive Health Centre, Vancouver, BC, Canada; St. Paul’s Hospital, Digestive Health Centre, Vancouver, BC, Canada

## Abstract

**Background:**

Patient comfort is a key quality indicator, associated with patient satisfaction and adherence. To effectively audit and improve comfort in endoscopy, comfort is measured using a standardized tool and compared against a benchmark. The St. Paul’s Endoscopic Comfort Score (SPECS) is a validated tool recorded for all endoscopic procedures at St. Paul’s Hospital.

**Aims:**

To establish a SPECS benchmark correlating to acceptable patient comfort during esophagogastroduodenoscopy (EGD) and colonoscopy.

**Methods:**

Between April 1^st^, 2025, and August 1^st^, 2025, three stakeholder focus groups (patient, nurse, and physician) were conducted to determine a SPECS threshold that reflect an acceptable level of discomfort during EGD and colonoscopy. Retrospective quantitate data were also collected from procedures performed at St. Paul’s Hospital between January 1, 2021, and December 31, 2023. Using the focus group-derived SPECS threshold, we calculated the proportion of comfortable procedures performed by each physician. Potential benchmarks were explored using three approaches: (1) Percentile-based benchmark - the physician at the 75th percentile sets the benchmark, (2) Average of high performers - mean of the top 25% physicians sets the benchmark, and (3) Achievable Benchmark of Care (ABC), which adjusts performance by procedural volume.

**Results:**

Fourteen physicians, ten nurses, and four patients participated in focus groups. Each group independently communicated that a SPECS of 0, 1, 2, or 3 would correspond to an acceptable level of discomfort, for both EGD and colonoscopy. A total of 23,585 procedures were analyzed (6,180 EGDs and 17,405 colonoscopies). The number of procedures per physician ranged from 184 to 782 for EGD and 355 to 1,840 for colonoscopy. At the 75th percentile, 95.94% of EGDs and 96.27% of colonoscopies had SPECS <4. The mean proportion of comfortable EGDs and colonoscopies among the top 25% of physicians was 96.76% and 96.78%, respectively. The ABC values were 96.85% for EGDs and 97.07% for colonoscopies.

**Conclusions:**

EGD and colonoscopy are generally well-tolerated procedures. All three benchmarking methods generated similar results, suggesting that over 96% of procedures should have a SPECS < 4. This benchmark can be applied at a site or physician level. Future research is needed to determine generalizability of these results to other centers.

**Funding Agencies:**

None

